# Functional Mapping before and after Low-Grade Glioma Surgery: A New Way to Decipher Various Spatiotemporal Patterns of Individual Neuroplastic Potential in Brain Tumor Patients

**DOI:** 10.3390/cancers12092611

**Published:** 2020-09-13

**Authors:** Hugues Duffau

**Affiliations:** 1 Department of Neurosurgery, Montpellier University Medical Center, 34295 Montpellier, France; h-duffau@chu-montpellier.fr; Tel.: +33-4-67-33-66-12; Fax: +33-4-67-33-69-12; 2 Institute of Functional Genomics, INSERM U-1191, University of Montpellier, 34298 Montpellier, France

**Keywords:** awake mapping, direct electrostimulation, functional neuroimaging, human connectome, neural networks, neuroplasticity, transcranial magnetic stimulation

## Abstract

**Simple Summary:**

Beyond intraoperative electrostimulation mapping in awake patients (the gold-standard for brain tumor surgery), non-invasive FNI/TMS can be repeated before and after resection(s). These techniques enable longitudinal investigation of neural reorganization, especially in low-grade gliomas, which can generate neuroplasticity in patients with usually no or slight neurological deficits, despite neoplasm within “eloquent” structures. Here, data gained from perioperative FNI/TMS mapping methods are reviewed, in order to decipher mechanisms underpinning functional cerebral reshaping induced by the tumor and its possible relapse, (re)operation(s), and postoperative rehabilitation. Heterogeneous spatiotemporal patterns of rearrangement across patients and in a single patient over time were evidenced, with structural changes as well as modifications of intra-hemispheric and inter-hemispheric functional connectivity. Such various fingerprints of neural reconfiguration were correlated to different levels of cognitive compensation. Serial multimodal studies exploring neuroplasticity might lead to new management strategies based upon multistage therapeutic approaches adapted to the individual profile of functional reallocation.

**Abstract:**

Intraoperative direct electrostimulation mapping (DEM) is currently the gold-standard for glioma surgery, since functional-based resection allows an optimization of the onco-functional balance (increased resection with preserved quality of life). Besides intrasurgical awake mapping of conation, cognition, and behavior, preoperative mapping by means of functional neuroimaging (FNI) and transcranial magnetic stimulation (TMS) has increasingly been utilized for surgical selection and planning. However, because these techniques suffer from several limitations, particularly for direct functional mapping of subcortical white matter pathways, DEM remains crucial to map neural connectivity. On the other hand, non-invasive FNI and TMS can be repeated before and after surgical resection(s), enabling longitudinal investigation of brain reorganization, especially in slow-growing tumors like low-grade gliomas. Indeed, these neoplasms generate neuroplastic phenomena in patients with usually no or only slight neurological deficits at diagnosis, despite gliomas involving the so-called “eloquent” structures. Here, data gained from perioperative FNI/TMS mapping methods are reviewed, in order to decipher mechanisms underpinning functional cerebral reshaping induced by the tumor and its possible relapse, (re)operation(s), and postoperative rehabilitation. Heterogeneous spatiotemporal patterns of rearrangement across patients and in a single patient over time have been evidenced, with structural changes as well as modifications of intra-hemispheric (in the ipsi-lesional and/or contra-lesional hemisphere) and inter-hemispheric functional connectivity. Such various fingerprints of neural reconfiguration were correlated to different levels of cognitive compensation. Serial multimodal studies exploring neuroplasticity might lead to new management strategies based upon multistage therapeutic approaches adapted to the individual profile of functional reallocation.

## 1. Introduction

The main purpose in glioma surgery is to optimize the extent of resection (EOR) while preserving or even improving the quality of life (QoL). Overall survival is significantly prolonged when a total or, if functionally feasible, a supramarginal resection (with a security margin beyond the Magnetic Resonance Imaging (MRI) signal abnormality) can be performed, both in low-grade gliomas (LGGs) [1,2] and high-grade gliomas (HGGs) [3]. Based upon these oncological considerations, image-guided resection was increasingly developed, relying initially on structural MRI and progressively on preoperative functional neuroimaging (FNI) (functional MRI (fMRI), magnetoencephalography (MEG), diffusion tractography imaging (DTI)) and transcranial magnetic stimulation (TMS) for surgical selection and planning [4,5]. Nonetheless, two decades afterwards, several limitations of these techniques were observed. A recent meta-analysis showed that fMRI has a low reliability, especially concerning language, with a sensitivity and specificity of 67% and 55%, respectively [6]. Moreover, fMRI cannot distinguish compensable cortical structures that can be removed versus critical areas that should be surgically spared. This drawback may lead to the under-selection of patients for surgery and can increase the rate of partial resection due to concern for damage of cortical sites deemed eloquent by fMRI [7]. Regarding DTI, beyond its high false-positive rate evidenced by an international tractography investigation [8], due to a marked inter-algorithm variability, especially in glioma patients [9], this technique is intrinsically a structural imaging modality not able to map the function of white matter (WM) tracts. Although combined MEG and TMS can provide an actual assessment of the neuronal activity at the cortical level (not based on neuro-vascular coupling as fMRI), they cannot directly investigate subcortical connectivity. Moreover, navigated repetitive TMS (rTMS) has only an 81.6% sensitivity, 59.6% specificity, 78.5% positive predictive value, and 64.1% negative predictive value for preoperative language mapping in brain tumor patients [10]. Therefore, preoperative FNI and TMS are currently not sufficient for efficient and reliable detection of cortical hubs and WM pathways critical for neural functions [11].

Consequently, to optimize the onco-functional balance by taking account of the considerable structural–functional variability across glioma patients, a paradigmatic shift emerged, with the development of intraoperative functional mapping-based surgical resection—tumor removal being pursued up to essential brain structures [12]. The principle is to identify and preserve the eloquent neural networks subserving movement execution and control, visuospatial function, language, cognition (e.g., attention, semantics, or executive functions), and behavior (e.g., theory of mind), by means of direct electrostimulation mapping (DEM) in awake patients, at both cortical and axonal levels [13]. This original philosophy resulted in a significant increase of survival and a decrease of disabling persistent deficits, including for gliomas within structures previously considered inoperable [14]. Such a new concept in neuro-oncology, which consists of studying the individual neural dynamics first (not the tumor), is also based on the understanding of the phenomenon of neuroplasticity, i.e., functional reshaping generated by brain injury, especially when the progression of disease is slow as in LGGs [15].

Here, the goal is to review the contribution of non-invasive perioperative FNI and TMS mapping, which offer the opportunity to investigate the mechanisms underlying neural plasticity not only before tumor surgery but also after resection(s), in order to integrate the potential of individual reorganization in the management of LGG patients and to elaborate new therapeutic strategies based on a tailored multistage approach.

## 2. The Concept of Neuroplasticity in a Meta-Networking Framework of Brain Processes

Contrary to what has been taught for more than one century, neural processes are not sustained by a rigid brain architecture founded on hyper-specialized sites (each discrete site being supposed to correspond to one specific function according to a traditional localisationist dogma) but rather by delocalized and resilient cerebral circuits—even though some areas as visual, auditory, or motor cortices are more specialized than higher order cognitive regions [13,16]. These large-scale networks underpinning conation, cognition, and emotion are composed of multiple cortical areas distributed over both hemispheres, interconnected by WM fibers that allow various patterns of synchronization resulting in brain functions: This view led to the concept of the functional connectome [13]. To explain the human propensity to learn complex abilities and to produce creative behaviors, a meta-networking theory of neural functions has recently been introduced, namely, an organization relying on networks of networks [16]. This principle is based on a perpetual succession of equilibrium states made possible thanks to interactions both within each neural network and between these functional systems, enabling a behavior adapted to moment-to-moment environmental demands. This dynamic framework with continuous changes of intra- and inter-circuits’ spatiotemporal integration can lead to long-lasting modulations of network properties, including use-dependent neuroplasticity. Plasticity can be defined as the brain’s ability to adapt to internal or external stimuli, and/or injuries. This major potential of cerebral reallocation occurring along the lifespan has many implications in physiology (for development, learning, or creativity) and in brain-damaged patients, since it may allow some degrees of post-lesional neurological recovery [16].

Interestingly, the neuroplastic potential is greater when the temporal pattern of the brain insult is slow, as in LGGs, explaining why patients with an LGG, even in so-called “eloquent” regions, usually experience no or only mild neurological disturbances (the diagnosis is generally made because of seizures) [17,18]. Furthermore, numerous surgical series reported a compensation after removal of LGGs involving structures classically deemed as “inoperable”, with a return to a normal familial and socio-professional life. Recovery was observed following glioma resection in Broca’s area, Wernicke’s area, Rolandic area, or the insula [15,17,19]. By achieving surgery made possible thanks to preoperative individual plastic phenomena, beyond preservation of QoL, the EOR was also maximized, with a higher rate of (supra)marginal resections, both in LGGs and HGGs [1,3]. Therefore, the principle of “prophylactic” surgery was introduced, proposing an earlier resection, including in incidentally discovered LGGs, i.e., when the potential for plasticity is greatest because the patients are not yet symptomatic [20,21].

In this connectome-based surgical resection, the needs of the patient and his/her family should be accurately defined before surgery, based on his/her lifestyle (job, leisure, or hobbies) and environment, in order to select the optimal tasks enabling a reliable intraoperative DEM and cognitive monitoring in awake patients [22,23]. Tasks should be adapted to the proximity of the cortical epicenters (e.g., complex bimanual movement close to the perirolandic cortex if the patient wants to preserve a high degree of dexterity [24]; or semantics near the dorsolateral prefrontal cortex to preserve higher-order cognitive functions [25]) and to the proximity of the WM tracts underpinning the functional systems [13,26]. For example, verbal and non-verbal semantic processes must be mapped regardless of the tumor location, since the inferior fronto-occipital fasciculus should represent the deep functional limit of the resection in all cases, due to its critical role in noetic consciousness and metacognition (knowing of knowing) [27,28]. A probabilistic atlas of functional plasticity derived from intraoperative DEM data on 231 patients who underwent LGG surgery evidenced that beyond the huge potential of cortical remapping (except regarding primary unimodal areas), subcortical connective WM tracts represented the main limitation of compensation [29]. Another atlas of essential nodal architecture of the connectome was also elaborated, reporting a normalized dataset of 1821 cortical and subcortical functional DEM-driven responses collected in awake LGG patients, and revealing the critical structures in 16 functional domains [30]. These atlases may be helpful for preoperative planning of glioma resection, to distinguish compensable areas hat can be removed from critical areas that must be spared.

Yet, the actual pathophysiological mechanisms underlying neuroplasticity at the individual level are still poorly understood. With the ultimate goal to predict before surgery how the brain will be able to dynamically reshape (or not) according to the EOR achieved at the expense of interactive cortico-subcortical networks in a given patient at this moment, perioperative non-invasive FNI and TMS mapping may provide unique information based upon longitudinal investigations, complementary to the intrasurgical DEM data, which can be collected only in real time within the operating theater.

## 3. The Contribution of FNI and TMS Mapping to Investigate Individual Mechanisms of Neuroplasticity before LGG Surgery

### 3.1. Preoperative Structural Changes in Glioma Patients

From a morphological perspective, changes in the macrostructure of the cortex in reaction to the glioma’s growth were evidenced using voxel-based morphometry. Indeed, insular LGG may generate a significant increase of the gray matter volume in the contra-lesional insula, supporting the hypothesis that homotopic reshaping could be a physiological foundation for functional compensation [31]. This recruitment of the contra-hemispheric homologous area might be made possible via the corpus callosum [32]. A greater gray matter volume of the left superior parietal cortex was also observed in patients with a right frontal tumor compared to healthy controls (HCs) [33]. Interestingly, the superior parietal cortex belongs to the control cognitive network, and such structural changes were correlated with executive functions in brain tumor patients [32]. Thus, the structural effect of tumor invasion may extend beyond the affected region, in a network-based reorganization (here, within the control cognitive network) underlying neuroplastic mechanisms. Interestingly, patients with hemispheric LGG involving language circuits can also exhibit an increased gray matter volume in language-cerebellar regions: Therefore, the cerebellum could be implicated in the process of post-lesional neuroplasticity [34]. At the ultrastructural level, these macroscopic changes might be underpinned by an increase in cell size or spine density, and neural or glial cell genesis [35,36].

Conversely, at the subcortical level, glioma migration seems to alter the integrity of the WM fibers, as evidenced by means of DTI that shows reduced fractional anisotropy values in tracts affected by the tumor [33]. This insult of the structural connectivity is correlated with a decline of cognitive functions, confirming that axonal bundles represent a major limitation of neuroplasticity. For example, invasion of the right superior longitudinal fasciculus is correlated to a visuo-spatial deficit [37], invasion of the left inferior fronto-occipital fasciculus to a semantic deficit [38], or invasion of the right cingulate to a mentalization deficit [39]. This low potential of structural reconfiguration and rewiring within the function-specific WM pathways [40] may indirectly participate in the compensatory changes of the intra- and inter-system functional connectivity at the level of the whole meta-networking brain.

### 3.2. Functional Redeployment and Correlation with Degree of Neurological Compensation

The brain can be analyzed by means of FNI as a series of temporally coherent circuits, with both adjacent and remote regions functioning in concert [41]. A large amount of data comparing glioma patients with HC using functional MRI (fMRI) showed distinct profiles of tumor-induced cerebral reorganization, both in the areas infiltrated by the glioma and in distal areas, supporting an effect of the tumor across a wide network and not only locally. By studying the functional connectivity (FC) that enables the measurement of how cerebral areas coactivate together over a given time frame [42], a decrease of FC in well-established networks with an increase in atypical FC patterns was reported [41,43,44]. This was observed for different functions, such as movement, language, visuo-spatial, and executive functions. Beyond the classical task-based fMRI, resting-state fMRI (rsfMRI) emerged as a prominent technique to investigate the intrinsic activity of cortical areas in an individual at rest [45].

#### 3.2.1. Preoperative Reorganization of the Motor Network

Presurgical task-based/rsfMRI [46] and TMS [47,48] in glioma patients have first supported a local rearrangement of cortical motor representations, made possible thanks to a more complex and dynamic organization of the primary motor cortex than previously thought [49]. However, such a potential of local reshaping has limitations, since the motor system serves as a unimodal output, and then belongs to the “minimal common brain” (the neural structures with a low plastic potential) [50]. Therefore, further mechanisms of compensation also rely on intra-hemispheric recruitment of secondary motor areas [46,51,52]. Indeed, movement control is sustained by a large-scale network, which implies notably the supplementary motor area (SMA); the premotor areas, including the “negative motor regions”, which elicit arrest of movement during DEM [24]; and the parietal lobe [53,54]. Moreover, involvement of contra-lateral homologous areas can be helpful regarding the neuronal computations necessary for movement planning and execution, owing to a modulation of the inter-hemispheric balance between lesioned and healthy hemisphere motor activation [46]. This concept of homotopic plasticity is based upon the recruitment of latent pathways or an upregulation of underused contra-hemispheric networks in normal conditions [55], in addition to glioma-induced changes in the transcallosal inhibition and inter-hemispheric competition, as already reported in stroke [56,57]. Several studies evidenced preoperative recruitment of the primary motor cortex and premotor cortex in the non-lesional hemisphere [58], with a possible reduced inter-hemispheric FC in comparison with intra-hemispheric connectivity in some patients [59,60] and a decreased ipsi-lesional FC in the sensorimotor network [61]. By achieving hierarchical measures of functional integration in LGG patients, inter-hemispheric integration during contra-lesional (but not ispi-lesional) hand movement was significantly stronger than in HC [62].

Although correlations between FC and neuropsychometric evaluations are scarce [51,63], the degree of motor compensation seems to be correlated to the pattern of reshaping. Reduced FC in the motor network is associated with motor deficit. Higher mean connectivity in tumor patients compared with HCs supports the hypothesis that the brain adapts to neoplasm progression by distributing functions across circuits, increasing FC to compensate lesion growth. Failure of redistribution across hemispheres is related to motor weakness, with a significant reduced inter-hemispheric FC between the ipsi-lesional primary motor cortex and the contra-lateral primary motor cortex and premotor areas in paretic patients [46,51]. A greater decrease of FC was observed in HGGs in comparison with LGGs [59], confirming that the plastic potential is correlated with the kinetics of the lesion [17].

#### 3.2.2. Preoperative Reorganization of the Language Network

Regarding language, a reduced within-language circuit FC with increased inter-network FC was observed in left frontal gliomas [64]. Wang et al. performed a presurgical fMRI language mapping (using naming task) in 43 patients with brain tumors at different locations. Although language areas were intact in right tumors, for left tumors, they noted a decreased FC in the classical left language network with an increased right FC connectivity, especially with a compensatory recruitment of the right inferior frontal gyrus in left frontal gliomas [65]. Li et al. also detected in left glioma a transfer of the function to the right healthy hemisphere, especially to the right inferior frontal gyrus [66], and a reduced activity in the lesioned hemisphere [67]. Briganti et al. [68] evidenced a global intra-hemispheric and inter-hemispheric reduced FC of the language circuit, in particular concerning the posterior connectivity of the left temporo-parietal junction, in patients with left gliomas performing a verb-generation task compared with HC. Using navigated TMS-based preoperative language mapping in 20 patients bearing a tumor in the dominant hemisphere, Raffa et al. [69] found a tumor-induced intra-hemispheric plasticity of language cortical areas. Furthermore, essential language regions were also evidenced by rTMS within the right hemisphere in tumor patients, particularly in the right inferior frontal gyrus [70]. Concerning syntactic processing, a correlation between network reorganization and agrammatic comprehension versus normal syntax was demonstrated using fMRI in glioma patients. Kinno et al. showed changes in the relative contribution of the three syntax-related circuits, with overactivation of the circuit involving the left lateral premotor cortex, while the circuit involving the left ventral frontal cortex was underactivated [64,71]. 

Functional reshaping of cerebro-cerebellar networks in left hemispheric glioma involving language areas was also evidenced by rsfMRI, with modifications of the activity in language-related cerebellar areas [34]. Interestingly, different patterns of neuroplasticity based upon various mechanisms of FC changes were correlated with different levels of cognitive disturbances: Cerebellar activity in regions with increased alteration was significantly associated with language (especially naming and comprehension scores) and MMSE Mini Mental State Examination scores [34]. Therefore, Yuan et al. [72] concluded that individual language processing investigated in patients with a left hemispheric tumor using rsfMRI relied on large-scale, bilateral, cortical-subcortical, and cerebro-cerebellar circuits with various network reallocation mechanisms underpinning the different levels of language worsening in LGG and HGG patients.

#### 3.2.3. Preoperative Reorganization of Other Networks

Regarding visuo-spatial function, Liu et al. [73] reported 17 patients with a temporal glioma (10 right and 7 left) in whom rsfMRI was achieved. Compared with HC, a significant increase of the amplitude of low-frequency fluctuation (ALFF) was observed in the contra-lesional homotopic mesiotemporal structures (hippocampus and parahippocampal gyrus), with changes in the FC between the left mesiotemporal regions and the left inferior temporal gyrus (increased FC) as well as between the left mesiotemporal regions and the left inferior frontal gyrus (decreased FC). The intrinsic regional activity in the hippocampus and parahippocampal gyrus was negatively correlated with the visuospatial scores [73].

Executive functions, which include working memory and mental flexibility, engaged during cognitively demanding tasks (e.g., reasoning, problem-solving, or decision-making), are underpinned by the executive control network (ECN), composed of fronto-parietal nodes including the dorsolateral prefrontal cortex and posterior parietal cortex [74]. By using rsfMRI in 37 patients with frontal glioma (16 left, 21 right) compared with HCs, Liu et al. [33] observed, in addition to a contra-lateral structural reorganization (see above), a functional recruitment of the contra-lesional ECN, especially of the superior parietal cortex (SPC), which seemed to play a pivotal role in the functional compensation of executive functions. In the right frontal tumor, an increased ALFF was noted in the left SPC, with a higher FC between the left SPC and the dorsomedial prefrontal cortex as well as between the left SPC and the contralateral SPC. Furthermore, increased ALFF in the left SPC within the ECN was positively correlated with executive functions [33]. Lang et al. [75] showed in 16 glioma patients who underwent preoperative fMRI during a working memory task (n-back) that a greater FC of the SPC within the tumor-affected hemisphere was correlated with lower-fluid cognition. Similarly, by using presurgical rsfMRI in 12 glioma patients, compared with 12 HCs, Maesawa et al. [76] reported a decrease in the left ECN FC strength and an increase in the right contra-lesional ECN. Interestingly, left ECN FC was correlated with attention, whereas right ECN FC was correlated with spatial memory scores.

The default-mode network (DMN), composed of bilateral and symmetrical hubs distributed across the ventromedial prefrontal, posteromedial, and inferior parietal, as well as the lateral and medial temporal cortex, is a critical circuit that deactivates during goal-directed tasks and which is involved in the monitoring of internally generated processes, e.g., autobiographical memory recollection, self-monitoring, and internal and external cognition [41,77,78]. Several series using preoperative rsfMRI in tumor patients noted a reduced FC across DMN areas in comparison with HC [76,79,80]. This difference was greater with left than right tumors, with parietal than frontal tumors, and with HGGs than LGGs [81,82]. Indeed, these changes in connectivity distributions were in part related to glioma grading [44], as supported by the fact that the DMN lateralized to the contralateral side in LGGs but not in HGGs [83]. Maesawa et al. found a significant correlation between resting-state DMN FC and cognitive functions, such as attention, working memory, and intelligence quotient [76]. Therefore, as demonstrated by combining neuropsychological evaluation and graph theoretical network analysis of rsfMRI, disturbed small-worldness property could be responsible for cognitive disturbances [84], especially in patients with frontal LGGs [85]. This is in agreement with MEG Magnetoencephalography studies, which found non-focal alterations in resting-state oscillatory brain activity in LGG patients [86], with correlations between an increase of the relative power in the theta and lower alpha band and worsened executive functions [87].

Finally, the salience network (SN), composed of the anterior insula and the anterior cingulate cortex, plays a pivotal role in switching the condition of activation and deactivation between the ECN and DMN [88]. In agreement with the meta-networking theory of brain processing, advanced cognitive functions rely on both within-network and between-network interactions among the distributed large-scale neural circuit [16]. In 13 patients with frontal glioma compared with HCs, beyond modifications of the FC in the SN, Liu et al. also evidenced increased strength and a greater number of FCs across specific areas selected from the DMN, ECN, and SN [76]. Therefore, a new perspective on intrinsically connected networks may be considered to explain higher-level mechanisms of neuroplasticity in tumor patients. Indeed, other circuits can help compensate the role of the insula in the SN, a strategy referred to as variable neuro-displacement [54]. In wide delocalized networks, such as the SN, due to its strong interactions with other networks, the functional reorganization could be more distributed, with possible involvement of alternative functional circuits.

To sum up, before any treatment, gliomas generate globally (and not only focally) altered functional connectomics profiles, with various patterns of neural reorganization allowing different levels of cognitive compensation. Beyond previous hierarchical models [89], despite a critical role of peritumoral areas (especially evidenced by preoperative TMS), contra-lateral homotopic plasticity could be very quickly engaged when the amount of injury of a given cortical region is too large to be compensated by the surrounding cortex, as supported by a frequent increase of contra-hemispheric FC concomitant to the decrease of FC within the ipsi-lesional damaged network. Indeed, although functional compensation was observed in the vast majority of studies, as long as the contra-lesional FC was maintained (see above), series with reduced long-distance FC reported a high rate of neurological deficits [90]. Furthermore, cross-network integration might also play a role, particularly for more widely distributed functions, increasing the variability in patterns of neural reconfiguration.

### 3.3. Preoperative Prediction of Postoperative Functional Outcomes Based on FNI/TMS

While scarce, suggestive evidence of presurgical network rearrangements predictive of transitory or permanent postoperative deficits were reported.

Regarding motor function, a transient global reduction in spontaneous movements contra-lateral to the operated side, with varying degrees of severity, occurred within a few weeks after the resection of a region activated in the posterior part of the SMA (SMA proper) on preoperative motor fMRI [91]. Concerning language, a transitory slowness in spontaneous speech, ranging from a complete mutism to a less severe speech impairment, which resolved in a few weeks, was observed when the activation in the SMA of the dominant hemisphere for language on presurgical fMRI was resected [92]. Lang et al. also showed that a lower preoperative FC in the parietal region contralateral to the tumor was predictive of poor neurocognitive outcomes 1 month following surgery [75]. These findings support the importance of a healthy hemisphere in recovery after resection of eloquent regions.

Beyond fMRI, presurgical resting-state whole-brain MEG recordings were also used to correlate the percentage of excised high FC voxels with postoperative motor, sensory, and language outcomes. Patients that had high FC tissue removed developed a higher rate of early and delayed postoperative worsening compared with patients that underwent resection of tissue with low FC [93]. More specifically, a linear correlation was found between the severity of auditory stimulus naming and syntactic impairment with the percentage of high FC areas removed [94]. Furthermore, Raffa et al. used preoperative TMS to investigate tumor-induced intra-hemispheric plasticity of language cortical sites: This allowed them to predict accurately postoperative persistent declines, particularly regarding the identification of false-eloquent areas [69].

Besides cortical reallocation, the integrity of the WM tracts was examined to predict postsurgical outcomes according to the preoperative tumoral invasion of the subcortical connectivity. Identifying presurgical language pathways using HARDI High angular resolution diffusion imaging q-ball fiber tractography was predictive of long-term language declines, especially when the dorsal stream was injured [95]. Using tractwise multiple regression analyses, the degree of tumoral infiltration of the main associative pathways was a significant predictor of postoperative deficits, e.g., subjective empathy and mentalization [96,97]. Indeed, WM bundles represent a major limitation of plastic potential. Knowing the risk of disconnection syndromes is critical to preserving postoperative QoL, underscoring the need to predict the EOR before surgery by using individual DTI combined with probabilistic atlases of functional resectability of LGG that take into account subcortical connectivity [50,98].

In summary, preoperative FNI/TMS mapping characterizing the mechanisms of neural redistribution generated by the tumor, notably by measuring the FC, can be helpful to counsel patients about the postsurgical risk and glioma resectability, and to tailor the surgical strategy accordingly. Further studies are needed to investigate more deeply the relationship between presurgical plastic reorganization and functional outcomes following resection, by also taking account of the various cognitive profiles before surgery, and the tumor features, i.e., its volume, its grade (LGG vs. HGG) [99], as well as its location. Indeed, a recent TMS study showed that the preoperative excitability of the motor eloquent areas (assessed using a cortical excitability score, which evaluates the number of abnormal inter-hemispheric resting motor threshold ratios) was significantly increased in isocitrate dehydrogenase wild-type gliomas [100]. Moreover, Vergani et al. [101] compared LGG in contact with the subventricular zone (SVZ) versus LGG not in contact with the SVZ. The extent of resection was smaller in LGG in contact with the SVZ, due to the infiltration of the WM tracts, which are the main limit of neuroplastic potential. To better understand correlations between patterns of network rearrangement and efficiency of postoperative recovery, post-resection FNI/TMS mapping should be more systematically achieved.

## 4. Functional Compensation after LGG Surgery: Postoperative and Longitudinal FNI/TMS Mapping Studies

Serial mappings are currently possible by means of non-invasive repeated FNI/TMS compared with neurocognitive scores before and after glioma resection. Indeed, in addition to standard neurological examination, neuropsychological assessment is another essential measure of the functional outcome related to brain plastic adaptation that provides a means of monitoring the progression of deficits over time in glioma patients [102].

### 4.1. Postoperative Reorganization of Motor Network

First, longitudinal motor mapping using repeated perioperative TMS evidenced a motor remodeling during the follow-up period, with a possible shift of hand representation site by more than 10 mm [47,48]. Recovery of transient postsurgical motor weakness was exclusively associated with cortical remapping, showing that despite the low potential of rearrangement of the sensorimotor area [50], long-term reorganization may occur after glioma surgery. Serial MEG studies achieved before surgery and at tumor relapse found a shift of the ipsi-lesional primary motor cortex occurring between MEG scans, correlated with the existence of a motor deficit and a longer period between both mappings [103]. However, a disengagement of the contra-lesional regions was noted at the second time point.

Conversely, using fMRI during self-paced movements of both hands performed before and after resection of LGG within the SMA, Krainik et al. [104] observed that (i) glioma progression generated presurgical under-activity in the adjacent SMA and over-activity in the contra-lateral SMA, (ii) postsurgical recovery of the transitory SMA syndrome was associated with recruitment of a contra-hemispheric medial and lateral premotor network comprising the healthy SMA and lateral premotor cortex, (iii) a correlation between the contra-lesional premotor recruitment and the percentage of resection of presurgical SMA activation, and (iv) shortened postsurgical recovery was correlated with increased preoperative modifications in activation levels. By achieving a serial hierarchical analysis of functional integration in these patients, although a symmetry of intra-hemispheric integration relative to movement was observed before resection of the SMA LGG, post-operatively, a modulation of the inter-hemispheric integration with the laterality of hand movement was found, with a preservation of the intra-hemispheric contra-lesional integration concomitant to a decrease in the intra-hemispheric ipsi-lesional integration, likely due to a surgical disconnection syndrome in the premotor region [62]. Similarly, Otten et al. reported two patients who had severe postoperative motor weakness: One patient recovered, with reconstitution of the FC in the motor circuit, including with the involvement of the contra-lateral hemisphere, while the other patient who experienced permanent deficit did not reconstitute the bilateral motor network [50]. Bryszewski et al. [105] also described activation of bilateral motor primary cortices and premotor areas, before and following surgery in 20 LGGs involving the sensorimotor system. Moreover, after resection, significantly greater intensity values of ipsilateral SMA were found in patients with a mild postoperative motor weakness compared with non-impaired patients. Conversely to LGGs, in 16 glioblastomas, Majos et al. [52] observed a postoperative trend of a decrease in the intensity and in the cluster size of activation of primary motor areas and premotor areas, both in ipsi-lateral and contra-lateral hemispheres.

**Figure 1:**
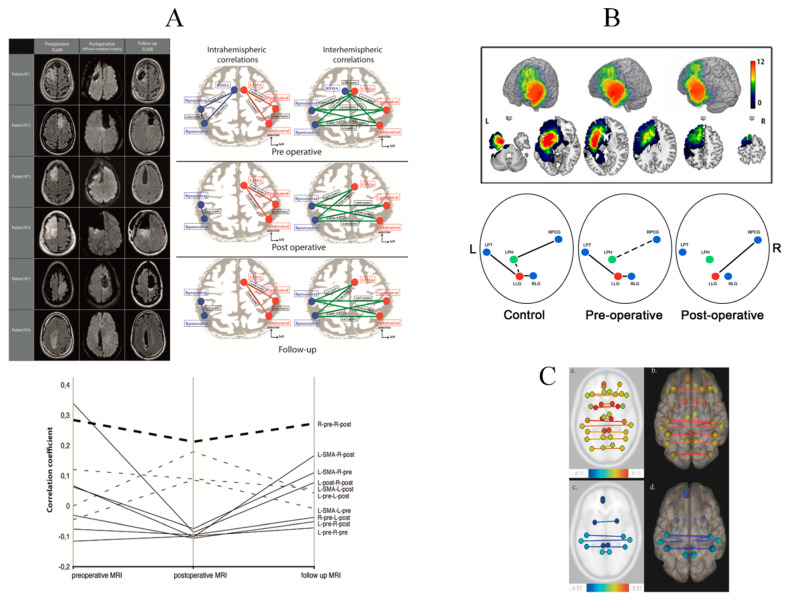
Serial fMRI before and after LGG resection (**A**): perioperative motor reorganization (from [106]). Left: Preoperative FLAIR-weighted MRI (left column), immediate postoperative diffusion-weighted MRI (center column) and delayed postoperative FLAIR-weighted MRI in 6 patients who underwent resection for an LGG involving the SMA. Right: Evolution of correlation maps within the resting-state sensorimotor network. Correlation coefficients between the sensorimotor network nodes, at both intra-hemispheric (left column) and inter-hemispheric (right column) levels, in the preoperative period (top), in the immediate postoperative period (center), and at 3 months’ follow-up (bottom). In both hemispheres, network nodes include the precentral region, the postcentral region, and the SMA; the SMA was resected on the lesional side and is therefore absent postoperatively. Lower: Longitudinal evolution plot of inter- and intra-hemispheric correlations in the sensorimotor network. The time course of all intra-hemispheric (dashed lines) and inter-hemispheric (continuous lines) correlation coefficients within the sensorimotor network are presented, showing a temporary decrease. (**B**): perioperative language reorganization (from [107]). Upper: Tumor locations in 32 patients who underwent awake DEM surgery for a left LGG. The sum of all tumor masks in pre-operative condition is displayed. The value in each voxel corresponds to the number of tumors in this specific location (range (0–12)). Lower: Schematic description of the main FC results. The three conditions are displayed: control subjects and patients in pre-and post-operative conditions. Green and red dots correspond respectively to LPH and LLG. Solid lines highlight evidence of FC. Dashed lines show slight FC close to significance or identified using one to one comparison. The FC of the LLG in pre-operative condition is similar to the one in control subjects. The LPH is connected to RPCG in controls and still slightly connected pre-operatively. In the post-operative condition, LPH loses all FC while LLG modifies its connectivity to connect to RPCG as LPH in controls. Abbreviations: RPCG = Right Precentral Gyrus; RLG = Right Lingual Gyrus; LPT = Left Planum Temporale; LPH = Left Parahippocampal gyrus; LLG = Left Lingual Gyrus. (**C**). (from [108]) FC variations in 82 patients who underwent awake DEM surgery for an LGG. Patients were scanned using rsfMRI successively before surgery (MRI-1), immediately after surgery, within 36 h following surgery (MRI-2), and three months after surgery (MRI-3). Comparison of MRI-1 and MRI-2 (a, axial section) (b, 3-D superior view) show a functional homotopy decrease (yellow to red lines). Comparison of MRI-2 and MRI-3 (c, axial section) (d, 3-D superior view) show a functional homotopy increase (blue lines).

Vassal et al. [106] performed rsfMRI preoperatively, during the immediate postsurgical period and 3 months following resection of 6 SMA LGGs (Figure 1A).

Data demonstrated a large-scale redistribution within the sensorimotor circuit, with a decrease of the inter-hemispheric FC in the postoperative period, when the patient exhibited a transitory neurological worsening, and with an increase again 3 months later when the patient recovered, with higher values than preoperatively, especially between the ipsilesional motor area and the contra-lesional SMA. Intra-hemispheric FC was also reduced during the immediate postsurgical period and returned to preoperative values 3 months later. Thus, inter-hemispheric FC could be a correlate of SMA syndrome recovery [109].

Furthermore, by achieving ALFF and regional homogeneity analyses on rsfMRI longitudinally before, immediately after surgery for hemispheric LGG, and 3 months later, Boyer et al. [109] evidenced subcortical modifications in addition to cortical changes, within the thalamus and the cerebellum. A transient hypo-activity was observed at their level 24 h following resection but no longer after 3 months. These results support a remote de-efferentation and de-afferentation generated by hemispheric resection: Such a transitory diaschisis might be involved in the immediate postsurgical worsening with subsequent recovery [109].

### 4.2. Postoperative Reorganization of the Language Network

Plastic mechanisms mediating perioperative language compensation were also investigated, especially in left LGG surgery. Using fMRI performed during a verb-generation task, Kristo et al. [110] reported left gliomas in which a greater activation of the left hemisphere was observed compared to the right one, both preoperatively and postoperatively. Five months following resection, the largest changes in the spatial patterns of activations were close to the left surgical cavity compared to the right homologous areas. Thus, perilesional activation of language regions in the proximity of glioma resection can account for cortical plasticity. In 16 other glioma patients, post-to-pre-resection intra-hemispheric rearrangement of activated voxels was noted in all cases, with the occurrence of new ipsi-lesional activation sites: Surgery in the dominant side shifted the activation from frontal towards temporal, while surgery in the non-dominant side shifted the activation from temporal towards frontal dominant regions [111]. Similarly, an MEG study using 12 left LGGs showed that FC 3 and 6 months after surgery was greater than preoperatively FC only in the peritumoral areas, within the alpha band, regardless of the glioma location [112].

Conversely, inter-hemispheric activations in the homologous language areas were evidenced, with an activation of the right “Broca’s area” in five frontal LGG patients without language disorders, and with an activation of the right “Wernicke’s area” in five temporal gliomas, both before and after surgery [113]. In a patient with resection for a left LGG near the “Broca’s area”, without language impairment, fMRI achieved before surgery and 3, 32, and 41 months after resection demonstrated a progressive right-sided activation of Broca’s and Wernicke’s areas [114]. Sarubbo et al. [115] identified a more complex pattern of redistribution on language fMRI performed following resection of a left LGG within the “Wernicke’s area”, with both intra- and inter-hemispheric compensatory reorganization. Using an fMRI naming task before and after resection of 32 left LGGs, Deverdun et al. [107] demonstrated that the pre-post surgical evolution of the left lingual gyrus (LLG) was correlated to the evolution of the naming task performance. Although, before surgery the LLG was connected to the left planum temporale and the homologous area (right lingual gyrus), following resection, the LLG was only connected to the right precentral gyrus (Figure 1B). Recently, using rsfMRI in 39 left LGGs, before and 3 months after surgery, neuroplasticity was detected in secondary areas, functionally connected to sites near the surgical cavity, with a greater connectivity of the left inferior temporal gyrus with the right and left inferior parietal lobule [14]. Beyond the language network itself, post-lesional recovery also depends on the integration and interaction of hubs not only within but also between multiple neural circuits, in agreement with the principle of variable neuro-displacement described in recovery from post-stroke aphasia [55]. Indeed, picture naming compensation relied on both the engagement of the semantic network and the fronto-parietal attentional network, as supported by an increased connectivity between the right superior parietal lobe and the right frontal operculum [116].

Such data emphasize the importance of a whole-brain approach during resting-state investigation in glioma patients, in a meta-networking account of neural functions. To examine longitudinal large-scale resting-state networks’ reallocation in LGG, rsfMRI was achieved in 82 patients before, immediately after awake surgery, and 3 months later, without a priori [108]. A transitory immediate postsurgical homotopic FC decrease was observed in cortico-subcortical supratentorial structures, with a functional homotopy increase at 3 months, especially in parietal lobes, cingulum, and putamen (Figure 1C). Similarly, by applying graph theory before and after LGG surgery, a disruption of the topological properties was detected, with an increase of the global efficiency while the local efficiency decreased [85].

Functional compensation is nonetheless possible only if the WM tracts are preserved, since in the majority of patients who do not completely recover after surgery, the persistent impairment is related to a disconnection syndrome due to damage of the subcortical fascicles, whatever the function, e.g., language or executive control [95,96,117,118,119]. Developments of new methodology that supports that blood-oxygenation level-dependent signals in WM may reflect neural activity in a resting state and under functional loading [120] could be of utmost interest to decipher the functional architecture of the axonal pathways in glioma patients.

## 5. Conclusions and Perspectives

Beyond intraoperative DEM in awake patients, which gives only an instantaneous reflection of brain processing during surgery, non-invasive FNI/TMS techniques, correlated with serial neurological and neurocognitive assessments, enabled to provide preliminary data regarding the mechanisms that underpin neural redeployment over time in reaction to a glioma’s progression and to its resection, both of which disturb endogenous local and global connectivity. Longitudinal mapping investigations evidenced heterogeneous spatiotemporal patterns of cortico-subcortical meta-network reorganization, founded on various combinations and balances between the recruitment of perilesional (tumor/surgical cavity) areas, the re-weighting of the ipsilesional large-scale intra-hemispheric FC between distant hubs, inter-hemispheric FC, and contra-lesional homotopic/heterotopic connectivity, as well as complex changes in the interaction across functional systems. These patterns might vary according to several factors, including tumor features and its time course (slow-growing LGG vs. rapid-growing HGG), the invasion of the subcortical connectivity (with a lesser degree of compensation when WM tracts are damaged), the function in question (with more bilaterally distributed circuits for higher-order cognitive functions), and the timing of the mapping (preoperative vs. immediate postoperative vs. late postoperative period). Indeed, it seems that the variability in the connectomal reconfiguration could be higher after than before resection. Of note, postsurgical rehabilitation per se can also induce functional rearrangement [22], as demonstrated by serial fMRI studies before and after cognitive rehabilitation programs following brain surgery [121].

At the ultrastructural level, experimental investigations of the cellular mechanisms mediating such a functional remodeling mainly relied on a competition between Hebbian synaptic plasticity [122], based upon the seminal idea that neurons firing together should wire together (founded on a balance between long-term potentiation and long-term depression) and homeostatic plasticity [123], which regulates the synapses to avoid instability and saturation (for a review, see [124]). In addition, animal studies have tried to compare one-stage versus multi-stage surgical resection, in order to explore the mechanisms underlying neuroplasticity according to the time course of the brain damage. Interestingly, for a similar extent of resection within the same brain location, animals with successive surgeries and rehabilitation following each surgical step recovered better than animals with one-stage ablation (for a review, see [17]). These data emphasize the role of temporal factors in recovery of function after brain damage, and support the concept of a multistage surgical approach in slow-growing LGGs.

**Figure 2:**
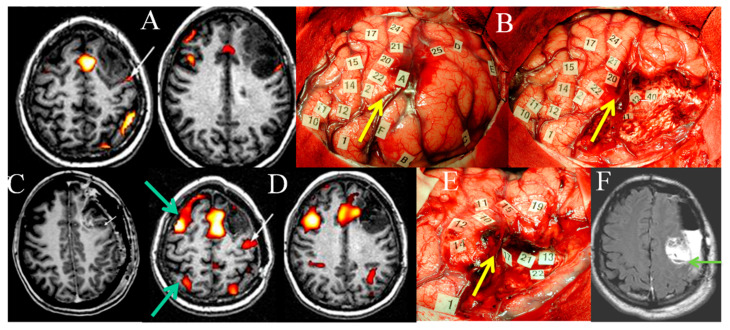
Illustration of remapping in the same patient demonstrated by serial FNI and DEM mappings. (**A**) Preoperative language fMRI in a patient without deficit, bearing an LGG within the left premotor area: language activation is near the posterior part of the glioma, within the precentral sulcus (white arrow). (**B**) Intrasurgical views before (left) and after (right) glioma resection, delineated by letter tags. DEM shows a reshaping of the functional maps, with a recruitment of perilesional language sites, enabling a subtotal resection with nevertheless a posterior residue due to the involvement of critical areas (number tags). The yellow arrow shows the precentral sulcus, demonstrating that it was not possible to remove the portion of the LGG involving the precentral gyrus. (**C**) Immediate postoperative enhanced T1-weighted MRI showing the tumoral residue (small arrow), in front of the precentral gyrus. (**D**) Postoperative language fMRI 4 years after the first fMRI, revealing a recruitment of the contra-lateral hemisphere (green arrows), and a posterior displacement of a left activation previously located within the precentral sulcus, which now shifted within the central sulcus (white arrow). (**E**) Intraoperative view during the second surgery, confirming the functional remapping, and making possible a more extensive glioma resection posteriorly, with no permanent deficit. Again, the yellow arrow shows the precentral sulcus, demonstrating that, this time, it was possible to remove a part of the glioma involving the precentral gyrus. (**F**) Immediate postsurgical axial FLAIR-weighted MRI (3 h after operation) confirming the increase of the extent of resection within the left precentral gyrus, thanks to functional reshaping (the green arrow shows the central sulcus). The patient resumed a normal social and professional life 3 months after reoperation, with no adjuvant oncological treatment. (The images are modified from [19,126])

The next step for patients suffering from glioma, particularly LGG, would be to use FNI/TMS mapping to predict, prior to any treatment, how and to what extent the brain is able to reorganize its meta-network following therapy in order to preserve QoL. The goal is to anticipate the dynamics of post-treatment neural reconfiguration according to the study of the individual potentials and limitations of neuroplasticity. Such knowledge should enable clinicians to tailor therapeutic strategies, not only for first surgery, but also reoperations and adjuvant oncological treatment, such as radiotherapy planned “a la carte” [125]. Following a seminal proof of concept that demonstrated the usefulness of serial fMRI before an initial resection and at LGG recurrence, permitting detection of remapping and opening the door of a second surgery—thanks to the confirmation of functional reshaping by means of intraoperative DEM in awake patient—the principle of a multistage surgical approach was proposed to improve EOR while avoiding neurological worsening (Figure 2) [126].

Several series have subsequently confirmed that optimizing LGG resection based on neuroplastic modifications was reliable [127,128], on the condition, nonetheless, to adapt the reoperation to the individual profiles of meta-network changes, which mainly depend on connectomal constraints [129]. It is critical to identify individual fingerprinting of functional connectivity that is behaviorally relevant [130], in order to predict further stage(s) of brain plasticity based upon FNI before and after a first resection. This may help to elaborate the best personalized long-term management dealing with the dynamic relationships between glioma behavior and neural adaptation, with the ultimate aim of optimizing the onco-functional balance in LGG patients who can now benefit from a life expectancy over 15 years. Typically, a further decrease of the ipsi-lesional FC compensated by an increase of the contra-lesional FC after (re)operation will facilitate subsequent surgery [129]. Of note, because radiation therapy may induce damage of the white matter tracts, which represents the main limitation of neuroplasticity, it has been proposed to defer irradiation for long-term preservation of cognitive function and QoL in LGG patients [125]. On the other hand, neuropsychological assessments performed after chemotherapy have evidenced that cognitive scores were similar or even improved, supporting that chemotherapy might facilitate neuroplastic mechanisms, especially when it induced a shrinkage of the glioma with a decrease of the tumoral infiltration along the WM tracts [131]. Indeed, in case of very diffuse LGGs, with a “gliomatosis-like” pattern, preventing to achieve a maximal resection due to a wide invasion of the subcortical connectivity, neoadjuvant chemotherapy can be administrated first, with the goal to generate a tumoral shrinkage, and then to optimize a subsequent resection [132].

The next challenge could be to guide neuroplasticity for one given patient at that time, for example, by using rTMS to potentiate cognitive rehabilitation with the aim of pushing the eloquent areas away from the tumor: Neuro-computational models [55,133] might be helpful to predict how to modulate the dynamic architecture of individual neural networks, within and between functional systems, at each step of therapeutic management.

To conclude, incorporating plasticity when planning resection is now a validated approach, which is ready for standard of care, based upon a large amount of data in the current literature. However, to better understand the individual patterns of dynamic structural and functional reorganization that will allow additional degrees of remapping following a first resection, further serial and multimodal studies are still needed in order to anticipate the next therapeutic stage tailored to each patient with a chronic LGG disease.

## Abbreviations

fMRI	functional MRI
rsfMRI	resting-state functional MRI
FNI	functional neuroimaging
DTI	diffusion tensor imaging
MEG	magnetoencephalography
TMS	transcranial magnetic stimulation
rTMS	repetitive TMS
DEM	direct electrostimulation mapping
LGG	low-grade glioma
HGG	high-grade glioma
WM	white matter
FC	functional connectivity
ALFF	amplitude of low-frequency fluctuation
EOR	extent of resection
SPC	superior parietal cortex
ECN	executive control network
DMN	default-mode network
SN	salience network
HC	healthy control
SMA	supplementary motor area
QoL	quality of life
SVZ	subventricular zone
